# The AI interviewer: multi-faceted evaluation of adaptive questioning by large language models

**DOI:** 10.1038/s41598-026-46517-7

**Published:** 2026-04-04

**Authors:** Anastasia Panfilova, Vadim Bolshev, Mikhail Mozikov, Vladislav Latynov, Alexander Vanin, Timofei Nestik, Veronika Nourkova, Arina Vlasova, Mikhail Kozin, Aleksandr Serohvostov, Elizaveta Tarasova, Sergey Nikolenko

**Affiliations:** 1 Institute of Psychology of the Russian Academy of Sciences, Moscow, Russia; 2 AI Research Institute, Moscow, Russia; 3 Saint Petersburg State University, Saint Petersburg, Russia

**Keywords:** Mathematics and computing, Psychology, Psychology

## Abstract

Large language models are increasingly deployed as adaptive interviewers in qualitative research and human-computer interaction, yet systematic evaluation of their interviewing behavior remains limited. We introduce a modular LLM agent for conducting semi-structured psychological interviews and present a controlled, multi-faceted evaluation protocol to assess interviewer quality across six state-of-the-art models: Claude Sonnet 4, Gemini 2.5 Pro, GPT-5 Chat, Grok 4, Qwen3-235B A22B, and DeepSeek Chat V3.1. The agent conducts adaptive interviews over 54 main questions spanning biography, family, interests, challenges, values, work, and health, deciding for each response whether a follow-up is warranted and generating tailored follow-up questions. To enable fair comparison, we standardize interview context using transcripts from ten baseline human interviews, execute all models under identical orchestration and prompts, and use a single LLM interviewee to eliminate human response variability. Expert psycholinguists evaluate interviewer behavior on five binary criteria: benevolence (empathic tone), necessity, context-awareness, openness, and justified skip (when follow-ups are unnecessary), annotating over 2900 items with high inter-rater reliability (Fleiss $\kappa$ 0.67–0.93). We complement human judgment with efficiency metrics (latency, questioning intensity) and linguistic profiling via morpho-syntactic and psycholinguistic features on the interview text. Results reveal systematic trade-offs: Gemini 2.5 Pro has the most empathic tone, GPT-5 Chat optimizes for speed and selective precision, Grok 4 achieves exhaustive coverage at the cost of latency and occasional over-contextualization, while Claude Sonnet 4 offers balanced versatility. Linguistic markers such as person pronouns, tense, intensifiers, or syntactic complexity align meaningfully with human judgments, suggesting that stylistic choices are aligned with perceived interview quality. DeepSeek’s format instability underscores the operational importance of schema compliance. Our reusable toolkit (prompts, orchestration code, annotation rubric) provides a foundation for principled deployment of LLM interviewers in psychological experiments, enabling researchers to match model capabilities to study goals and to audit agent behavior for empathy, appropriateness, and effectiveness.

## Introduction

Qualitative research in psychology, sociology, and human-computer interaction has long relied on semi-structured interviews to provide rich, contextualized data about human experiences, values, and behaviors. A skilled interviewer must balance structure with flexibility, following a predesigned protocol while at the same time adapting to individual responses through empathic, context-aware follow-up questions that deepen understanding without leading or overwhelming the respondent. This adaptive interviewing skill is difficult to scale: conducting hundreds of in-depth interviews requires substantial time, expertise, and resources.

The recent capabilities of large language models (LLMs) have opened a promising avenue: deploying LLM-based agents as adaptive interviewers. Early applications already include health check-ins, biographical life-story elicitation, and assessment of values, with case studies demonstrating feasibility. Still, there are two critical gaps that prevent an immediate switch to LLMs and generally impede progress toward principled, reproducible deployment.

First, we lack a reusable evaluation framework that operationalizes the qualities of good interviewer behavior. Existing assessments often rely on downstream task success (e.g., diagnostic accuracy) or generic user satisfaction, overlooking whether an agent’s follow-up questions are empathic, contextually grounded, warranted, and non-leading; all of these criteria are crucial for the qualitative methodology. Without such measures, practitioners cannot systematically compare models or audit agent behavior for deployment in sensitive settings like psychological research.

Second, cross-model comparisons in the literature are frequently confounded by variations in human respondents, orchestration details, and experimental conditions, making it unclear whether observed differences reflect the underlying LLMs or artifacts of study design. To produce fair performance benchmarking, we need controlled protocols that would isolate model behavior but still keep the overall methodology valid.

In this work, we address both gaps. We introduce a modular LLM agent for conducting adaptive psychological interviews, paired with a multi-faceted evaluation protocol. The agent conducts semi-structured interviews over a fixed battery of 54 main questions spanning biography, family, interests, challenges, values, work, and health. For each response, the agent decides whether a follow-up is needed and, if so, generates a tailored follow-up question. To enable fair cross-model comparison, we standardize three key elements:

- interview context, using transcripts from human baseline interviews with real respondents;
- orchestration, executing all models under identical prompts, guardrails, and retry logic;
- follow-up responses, answered by a single LLM interviewee to eliminate human variability.

Expert annotators then evaluate interviewer behavior using five binary criteria designed specifically for qualitative interviews: empathic tone (benevolence), necessity, context-awareness, openness (non-leading phrasing), and justified omission (when skipping a follow-up is appropriate). Beyond human judgment, we quantify efficiency (latency and throughput), questioning intensity, and linguistic style through morpho-syntactic and psycholinguistic features in Russian text. This joint analysis yields robust, interpretable contrasts among contemporary LLMs and provides actionable guidance for deploying interviewer agents in psychological experiments.

**Fig. 1:**
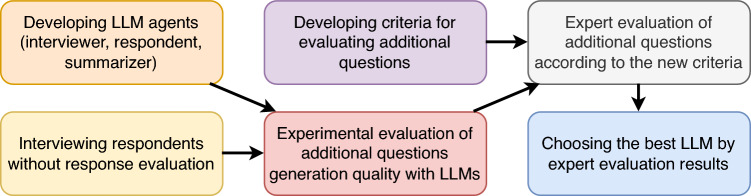
The structure of our study.

*Main contributions* The structure of our study is illustrated in Fig. 1. Our main contributions in this work include the following.

1. *An interviewer agent and orchestration recipe.* We present a modular agent that reasons about respondent answers, decides whether follow-ups are needed, and generates tailored questions under strict output schemas. The same prompts, guardrails, and retry logic are applied across all models to ensure comparability.
2. *A principled human evaluation framework.* We introduce five binary, face-valid criteria targeting interviewer behavior (empathic tone, necessity, context-awareness, openness, justified skip) and demonstrate high inter-annotator agreement on 2900+ annotated items, enabling reliable benchmarking.
3. *A controlled cross-model comparison.* Using identical baseline interviews and a fixed interviewee, we compare state-of-the-art LLMs on human criteria, efficiency, and questioning intensity, revealing consistent strengths, weaknesses, and style differences that matter for psychological interviewing.
4. *Linguistic profiling of follow-ups.* We provide corpus-level analysis of follow-up questions using morpho-syntactic counters and a Russian LIWC-like dictionary, surfacing model-specific markers (person pronouns, tense/aspect/mood, intensifiers, syntactic complexity) that align with human judgments.
5. *A reusable toolkit and rubric.* We release prompts, orchestration code, and annotation rubrics, aiming to standardize evaluation of LLM interviewers and lower barriers to adoption in psychological experiments and qualitative research.

Together, these elements form a compact methodology for developing and auditing LLM interviewers. Our findings suggest that current models differ not only in efficiency but also in their capacity to ask follow-ups that are empathic, warranted, and contextually grounded—properties central to high-quality interviewing that our rubric makes measurable and comparable. We discuss implications for study design, model selection, and safe deployment in psychological research settings, and outline limitations and directions for future work.

## Related work

### Simulating human behaviour with LLM-based agents

Early demonstrations showed that large language models (LLMs) can be embedded into agent architectures that produce believable day-to-day behaviours and social dynamics. Park et al. ^1^ introduced *generative agents* with memory, reflection, and planning modules, producing emergent social behaviour in a sandbox town. More recently, Park et al. ^2^ instantiated over 1000 person-specific agents derived from qualitative interviews and reported that these agents reproduced the participants’ responses on the general social survey nearly as reliably as the participants themselves 2 weeks later; they were also reported to generalize in personality and experimental replications. These case studies led to a surge in research on LLM-driven social simulation; we refer to recent surveys that map the space from individual to society-level simulation, evaluation protocols, and open challenges ^3–6^.

### LLMs as cognitive models and theory-of-mind simulators

A different line of work treats LLMs as *models of cognition*. Binz and Schulz ^7^ showed that fine-tuning LLMs on behavioural datasets can outperform classic cognitive models across decision-making tasks, suggesting the prospect of generalist cognitive models. This line culminated in *Centaur*, a foundational model of human cognition trained on *Psych-101* (trial-level data from over 10M choices across 160 experiments), which predicts human behaviour across domains and exhibits more human-aligned internal representations after fine-tuning ^8^. Complementary evaluation frameworks probe cognitive signatures in LLMs; for instance, Coda-Forno et al. ^9^ proposed *CogBench* to phenotype LLM behaviour using cognitive psychology metrics.

Within social cognition, comprehensive tests suggested that GPT-4 already matched or exceeded human averages on several theory-of-mind tasks (false belief, indirect requests) while failing in others (e.g., faux pas) and showing brittle dependence on phrasing ^10^; this study also emphasized that some theory-of-mind successes can reflect dataset priors or prompt artifacts rather than robust capacities. Overall, this line frames LLMs as useful *cognitive mirrors*: predictive of behaviour in many tasks, but not straightforwardly explanatory of human mechanisms.

### LLMs as virtual participants and predictors of experimental outcomes

Aher et al.’s “Turing experiments” first showed LLMs can replicate classic findings when prompted as multiple synthetic participants ^11^. Scaling this idea, Cui et al. ^12^ replicated 154 psychology experiments with GPT-4, recovering 76% of main effects but only 47% of interactions and with effect-size distortions, arguing for cautious use as a complement rather than a replacement for human subjects. Beyond replication, LLMs have been used to *predict* outcomes of social science field experiments at scale: Chen et al. ^13^ report $\sim$78% accuracy over 276 field experiments in economics and attempt to characterize boundary conditions (e.g., complex social issues). However, other researchers warn of confounding and emphasize explicit validity criteria when deploying LLMs as “participants” ^14,15^.

### Personality and psychological profiling

There is growing evidence that LLM representations encode information about perceived personality; even simple predictive models for personality can improve recommendations ^16,17^, but this direction has blossomed in the era of LLMs. Using GPT-3 embeddings of names, Cao et al. ^18^ predicted public perceptions of big five traits for hundreds of public figures with notable accuracy. Peters et al. ^19^ showed that LLMs can infer the user’s Big Five traits from free-form chats, outperforming prior supervised baselines. At the same time, psychometric evaluations highlight instability and rater disagreement when administering personality inventories *to* LLMs, and mixed temporal reliability across models^20,21^. Together, these findings suggest two distinct but related applications: (i) extracting perceived personality signals *from language about people* and (ii) instrumenting LLMs as profilers over user-generated text—each requiring distinct validity checks and safeguards. Recent research also shows that we can successfully induce personality traits into LLMs ^22^ and/or use an LLM’s personality profile to shape its behaviour, e.g., with respect to risk taking ^23^, and it is known that the personas impersonated by LLMs can significantly change their performance across a wide range of tasks ^24^.

### Moral judgement, social decision making, and bias

Several studies compare LLM and human performance in social judgement tasks. On established situational judgement tests, several LLMs equalled or surpassed human means already in 2024 ^25^. In moral reasoning, human evaluators sometimes rate LLM explanations as comparably (or more) moral or trustworthy than human ones, even rivaling expert ethicists in perceived expertise ^26^. On the negative side, several other assessments caution that LLM advice can amplify certain cognitive biases ^27^ and that moral judgement elicitation is fragile to methodology and prompting ^28^. Overall, LLMs can be strong performers in moral or social judgement benchmarks, but construct validity and robustness are still controversial.

### Clinical and mental health applications

In clinically oriented psychology, LLMs have been widely explored for assessment, triage, and therapeutic support ^29–36^. For example, Kim et al. ^37^ report LLMs outperforming medical and mental-health professionals in differential diagnosis of OCD vs. mimicking conditions under controlled prompts. At the same time, while researchers and practitioners often argue that psychotherapy and mental health care could be transformed by LLMs, they also emphasize risks such as harmful suggestions, overtrust, cultural bias, or sycophancy, and regulatory hurdles that could slow down adoption; recent evaluations highlight the need for domain-specific guardrails, disclosure, and clinical oversight ^37–39^. The emerging consensus is that LLMs are already promising assistive tools with a potential to become increasingly autonomous clinicians, and recent research in this area is helped by emerging standard datasets and benchmarks such as the CBT-Bench ^40^. Another important advantage is easy scaling: while one cannot have professional human psychologists analyze, e.g., social media for signs of mental illness, LLMs have already been adopted for such preliminary screening ^41–44^. However, although currently LLMs are already transforming mental health and psychology, many caveats still remain, and often articulated in recent analyses. For example, Schröder et al. ^45^ argue that LLMs should not be considered faithful simulators of human psychology, while Lin ^46,47^ argues against treating LLMs as population averages and instead frames them as linguistic simulators and cognitive models with specific validity conditions.

## Methods

This section describes the experimental design, agent architecture, annotation protocol, and statistical procedures used to evaluate large language models as adaptive interviewers in semi-structured psychological interviews. The full prompts and additional information are provided in the “Appendix”.

### Study design and materials

We conducted a controlled, replay-based, text-only evaluation to compare six contemporary LLMs acting as interviewers. The key unit of analysis was adaptive follow-up behavior rather than generation of the main interview script itself: at each turn, the model received the same main question and the same respondent answer, and we evaluated whether it decided to ask a follow-up and how it formulated that follow-up. This design eliminates between-participant variability by using a fixed set of baseline human interviews as input for all models, ensuring that differences in interviewer behavior can be attributed to the models themselves rather than to variations in respondents or initial conditions.

*Baseline interviews* We first collected ten standardized human interviews with real respondents. In these source interviews, a human interviewer asked only the 54 main questions from a fixed script, without following up with any adaptive questions. This constraint was essential: it created a standardized starting point where each LLM interviewer would receive identical initial responses to the same questions, allowing for controlled comparison. Each session was audio-recorded, professionally transcribed, and de-identified. These transcripts formed the base “main question + human answer” stream for all subsequent LLM interviewer evaluations.

*Interview protocol* The interview script consists of 54 main questions spanning seven key life domains: biography and background, family and relationships, interests and hobbies, formative life events and challenges, personal values and beliefs, current professional activity and career, and health and well-being. Questions were designed to invite open, narrative responses that would naturally prompt follow-up inquiry in a human-conducted interview. Here are some examples of main questions:

- “Tell me about your childhood and where you grew up,”
- “What are the most important values that guide your life decisions?”
- “Describe a challenging period in your life and how you handled it.”

The complete script is provided in “Appendix B”, and the original Russian version is available upon request.

*Experimental conditions* Each of the six LLM models served as the interviewer for all ten baseline transcripts, generating a total of 60 interview sessions (6 models $\times$ 10 transcripts). Within each session, the LLM interviewer processed the 54 main questions sequentially. For each main question, the model received the corresponding human answer from the baseline transcript and decided whether to ask follow-up questions. If a follow-up was asked, the ensuing interaction remained text-only: the question was answered by the LLM interviewee, the interviewer re-evaluated that answer, and it could ask another follow-up if the answer was still incomplete. This loop continued until the model returned NEXT_MAIN or the per-question follow-up cap was reached. This replay-based design ensured that all models evaluated the same initial responses under identical conditions.

All interactions were conducted in Russian and in text form, reflecting the language of the original interviews and enabling analysis of linguistic features specific to Russian morphology and syntax; no prosodic, acoustic, or visual cues were available to the models.

### LLM agents and orchestration

We implemented a multi-agent system with three specialized LLM agents coordinated by a state-machine orchestrator. The primary agent is the *interviewer*, which analyzes respondent answers, decides whether follow-ups are warranted, and generates targeted follow-up questions. Two auxiliary agents support the pipeline: an *interviewee* that provides standardized responses to follow-up questions (eliminating between-participant variability) and a *summarizer* that periodically compresses long-term memory to prevent context overflow. The logic of LLM agents is implemented as a state graph using the LangGraph framework, ensuring consistent execution throughout the workflow.

#### State representation and memory management.

At each turn of the interview, a shared state object maintains the following components:

- target_question: the current main question from the interview script;
- user_answer: the most recent answer under evaluation, either the human’s response from the baseline transcript (for main questions) or the LLM interviewee’s response (for follow-up questions);
- short_term_memory: a sliding window transcript capturing approximately the last 2000 characters of dialogue, providing immediate conversational context;
- reflection_notes: a structured, cumulative record of key facts about the respondent extracted throughout the interview, serving as long-term memory;
- meta_info: configuration settings including interview language and interviewer persona;
- reasoning: the structured output from the interviewer LLM containing analysis, decision rationale, and an optional follow-up question;
- decision: a binary decision marker (NEXT_MAIN or FOLLOW_UP) indicating whether to proceed to the next main question or ask a follow-up;
- tailored_question: the generated follow-up question (when applicable).

This dual-memory architecture, which combines short-term conversational context with long-term factual accumulation, allows the interviewer to maintain coherence across the full interview while staying within token limits.

**Fig. 2:**
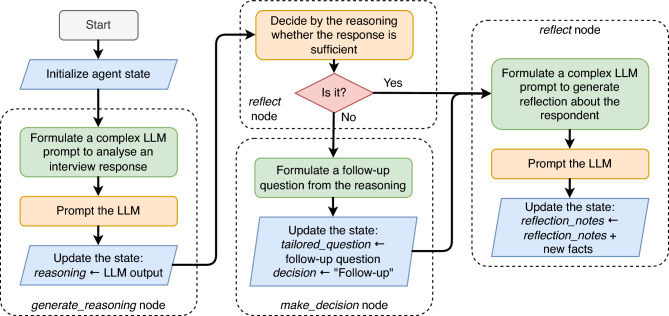
Flowchart of the LLM interviewer agent.

#### The interviewer agent

The interviewer agent operates through a four-node cycle for each respondent answer (Fig. 2):

1. *The *generate_reasoning *node*. It serves as the graph’s starting point, where the LLM generates a hypothesis on how adequate the respondent’s answer is. The agent makes a single LLM call that receives the current main question, the answer under evaluation, short-term conversational memory, and cumulative reflection notes. The prompt instructs the model to:
  - assess whether the answer already contains the key information required for a complete response to the main question;
  - provide a brief justification (3–4 sentences) for this assessment;
  - output a binary decision (“yes” for complete, “no” for incomplete); and
  - if the answer is incomplete, propose a single, specific, non-redundant follow-up question targeting the identified information gap.
  The prompt emphasizes several interviewing principles: follow-ups should be empathic and non-judgmental; they should build on information already provided rather than repeating questions; they should avoid leading or yes/no phrasing unless specifically warranted; and they should focus on obtaining missing information rather than excessive detail on topics already covered. The final response must strictly conform to the following format: an evaluation, a binary decision, and, if necessary, a clarifying question. This approach ensures consistency in responses and allows the prompt to be used in the interviewer agent’s logic to retrieve the decision and generated question at subsequent stages. The complete raw output is captured in the reasoning field. We note that in typical LLM agents, response evaluation and question generation tasks are usually done in separate LLM calls. We merge these tasks, substantially lowering query costs and the total time required to process an interview. Experiments (see below) validate this approach, as most contemporary LLMs successfully manage to do this combined task.
2. *The* make_decision *node*. This node is a logical router implemented as a conditional edge in LangGraph terminology. It parses the binary decision marker from the reasoning output and proceeds to one of two branches:
  if the extracted marker is “no” (the respondent’s answer is considered incomplete or insufficiently informative), control flows to generate_follow_up node;
  if “yes” (the answer is considered complete), control flows to the reflect node, where the agent analyzes the completed dialogue.
3. *The* generate_follow_up *node*. It is activated only after the make_decision node finds the answer to be incomplete, and it extracts a follow-up question for further interaction with the respondent. The node parses the reasoning field, extracts the question text, and stores it in a separate tailored_question field. Note that there is no new call to the LLM at this stage: this node only extracts existing information, saving operational costs and latency. Additionally, it assigns the FOLLOW_UP label to the decision field, which serves as a flag for the external system (orchestrator) to ask the respondent a follow-up question.
4. *The* reflect * node*. It serves as the final step in both logical branches, accumulating knowledge about the respondent, and initiates the second call to the LLM with a purpose to generate new data. Specifically, this node updates reflection_notes by extracting 1–2 new objective facts (maximum 15 words each) from the respondent’s current answer. The prompt instructs the model to avoid duplicating information already present in reflection_notes and to focus strictly on concrete, verifiable information rather than interpretations or subjective analysis.

#### The interviewee agent

**Fig. 3:**
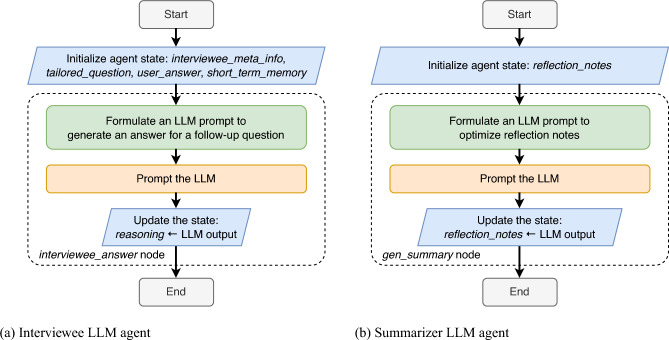
Flowcharts of auxiliary LLM-based agents.

To eliminate variability from human respondents when comparing models, all follow-up questions were answered by a single LLM interviewee built on GPT-4o (Fig. 3a). The interviewee agent operates through a single-node graph that constructs responses using:

- a fixed big five personality profile derived from the respondent’s pre-interview psychometric assessment;
- the current short-term memory providing conversational context; and
- the incoming follow-up question.

The prompt instructs the interviewee to respond in a manner consistent with the established personality profile, to maintain consistency with information already disclosed, to provide natural conversational responses in Russian, and to avoid contradictions with prior statements. This approach ensures that all interviewer models receive responses of comparable quality and consistency, isolating differences in interviewer behavior as the primary variable.

#### The summarizer agent

To prevent memory overflow during long interviews, a summarizer agent performs periodic compression of the reflection_notes (Fig. 3b). The generate_summary node is triggered every 10 respondent answers and makes a single LLM call with a compression prompt. The prompt requires at least 20% length reduction through deduplication and semantic merging of related facts, while strictly preserving core biographical and demographic information that may be referenced later in the interview.

#### Orchestrator logic

**Fig. 4:**
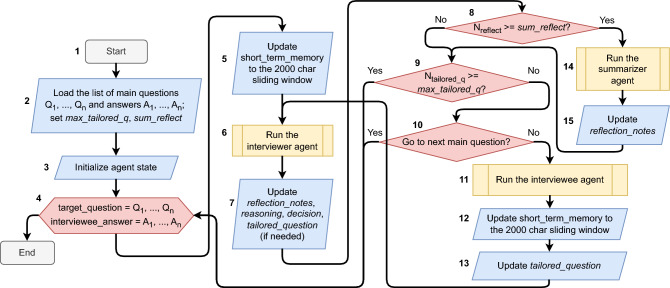
Flowchart of the orchestrator.

The orchestrator coordinates all agents through a state machine that processes each interview (Fig. 4). For each of the 10 baseline interviews and each tested model, the orchestrator goes through the following algorithm.

1. Initialize the agent state with empty memory structures (**3** in Fig. 4).
2. Iterate through the 54 main questions in order (**4**).
3. For each main question:
  - inject the corresponding human answer from the baseline transcript;
  - update the 2000-character sliding window of short-term memory (**5**);
  - run the interviewer agent cycle (**6**), update the fields of the agent state (**7**).
4. If decision = NEXT_MAIN (**10**), advance to the next main question (**4**).
5. If decision = FOLLOW_UP:
  - pose the follow-up to the LLM interviewee (**11**);
  - update the sliding window of short-term memory with the follow-up exchange (**12**);
  - re-run the interviewer agent to evaluate the interviewee’s response (**13**).
6. Continue the follow-up loop until the interviewer returns NEXT_MAIN or a per-question follow-up cap is reached (**9**; max_tailored_q is fixed at 5 across all models to prevent runaway loops).
7. Trigger the summarizer every 10 respondent answers to compress reflection notes (**8**, **14**, **15**).

This orchestration ensures that all models operate under identical constraints, prompts, and memory management policies, enabling fair comparison. Thus, a model could ask multiple successive follow-up questions within the same main-question turn, but never more than five.

### Models under test and API access

We evaluated six state-of-the-art LLMs in a single study window in late September–early October 2025: Claude Sonnet 4 (Anthropic), Gemini 2.5 Pro (Google DeepMind), GPT-5 Chat (OpenAI), Grok 4 (xAI), Qwen3-235B A22B (Alibaba Cloud), and DeepSeek Chat V3.1 (DeepSeek). All models were accessed through the OpenRouter unified API. OpenRouter was configured with automatic provider selection (i.e., no manual provider pinning), while system prompts, role instructions, and temperature setting (0.7) were kept identical across conditions. Only the underlying base model varied across conditions. For each API call, the orchestrator logged timestamps (start and end), raw latencies, and token usage (prompt and completion tokens). Aggregate costs per interview and effective cost per realized follow-up were computed from provider billing metadata via the OpenRouter reporting layer. Table 1 summarizes the exact model identifiers and the key orchestration settings used in the study.

**Table 1:** Reproducibility checklist for API access and orchestration.

Item	Setting used in the study
API access layer	OpenRouter unified API
Routing policy	Automatic provider selection; no manual provider pinning
Call window	Late September—early October 2025
Interviewer models	Claude Sonnet 4 (anthropic/claude-sonnet-4); Gemini 2.5 Pro (google/gemini-2.5-pro); GPT-5 Chat (openai/gpt-5-chat); Grok 4 (x-ai/grok-4); Qwen3-235B A22B (qwen/qwen3-235b-a22b-thinking-2507); DeepSeek Chat V3.1 (deepseek/deepseek-chat-v3-0324)
Temperature	0.7 for all interviewer models
Schema repair	Up to 3 constrained retries after validation failure
Follow-up cap	max_tailored_q=5 per main question
Short-term memory	Sliding window of approximately 2000 characters
Summarization trigger	Every 10 respondent answers

### Outcome measures

We recorded the following quantitative measures for each model.

1. *Latency metrics* total wall-clock time for completing all 10 interviews; average time per interview; average time per follow-up question generation (measured at API call boundaries).
2. *Questioning intensity* average number of follow-up questions per interview; average number of follow-ups per main question.
3. *Question characteristics* mean character length of follow-up questions; linguistic features (see below).

### Expert annotation

We have performed expert annotation as our human evaluation of the LLMs as helpers in psychological experiments.

#### Evaluation criteria

According to the above-mentioned approaches to assessing interview agents, we developed five binary criteria to assess interviewer behavior.

1. *Benevolence* the follow-up question demonstrates a positive, empathic tone, for example, by acknowledging the effort or courage required to share difficult information, expressing appropriate sympathy, or showing appreciation for the respondent’s openness.
2. *Necessity* the follow-up question is warranted given the completeness and detail of the current answer, i.e., the answer genuinely lacks information relevant to the main question that the follow-up could elicit.
3. *Context-awareness* the follow-up integrates information from prior responses in the same interview, demonstrating that the interviewer has been attending to and building upon earlier disclosures.
4. *Openness* the follow-up avoids “suggested answers” or leading phrasing, inviting free expression rather than constraining the response (e.g., avoiding closed yes/no questions unless specifically appropriate).
5. *Justified skip* used only when no follow-up question is asked. The decision to skip a follow-up is appropriate because the main answer already provides sufficient, complete information.

Detailed rubrics with positive and negative examples for each criterion are provided in “Appendix C” (“Criteria with examples”).

#### Annotation dataset and procedure

Three expert psycholinguists with training in qualitative research methods independently annotated the interviewer outputs. The annotation dataset consisted of

- 1658 LLM-generated follow-up questions evaluated on criteria 1–4 (benevolence, necessity, context-awareness, openness) and
- 1275 main-question turns where no follow-up was asked, evaluated on criterion 5 (justified skip).

For each item, annotators assigned a binary label (yes/no) indicating whether the criterion was met. Annotators were blind to model identity during evaluation. We computed per-model mean scores as the proportion of “Yes” responses across all items for that model and criterion.

#### Inter-rater reliability

To assess annotation consistency, we computed Cohen’s $\kappa$ for all three pairs of annotators and Fleiss’ $\kappa$ across all three raters for each criterion. Interpretation followed standard conventions: $\kappa > 0.80$ indicates “almost perfect” agreement, 0.60-0.80 means “substantial” agreement.

#### Linguistic profiling of follow-up questions

To characterize stylistic differences among models, we analyzed each follow-up question using computational linguistic features. The feature set consisted of:

- *Morpho-syntactic counts* (per question): total words (N_words), adjectives (N_adj), adverbs (N_adv), coordinating and subordinating conjunctions (N_cconj, N_sconj), personal pronouns by grammatical person (N_pronn_pers_first, second, third), verb morphology including tense (past, present, future), aspect (perfective, imperfective), mood (indicative, imperative), and voice (active, passive), syntactic tree statistics (mean, maximum, and minimum syntactic depth), repeated words within a question; average and maximum token lengths.
- *Intensifiers*, i.e., words that strengthen and give additional emotional context to the lexical element that changes (e.g., *very*, *extremely*, *absolutely*, *totally* etc.).

All features were extracted using morphological and dependency parsing together with dictionary matching. We used SpaCy to extract lemmas, part-of-speech tags, morphological features for inflected wordforms, and dependency-tree annotations in the Universal Dependencies format. Intensifiers were matched using a classical dictionary of Russian intensifiers available at https://ruslang.ru/intens_Dostoevsky.

#### Statistical analysis

All hypothesis tests were two-sided with significance threshold $\alpha = 0.05$.

1. *Human evaluation* for each criterion, we computed per-model mean proportions (“Yes” rates) and 95% confidence intervals using the normal approximation. To test for overall differences among models, we applied one-way ANOVA to per-interview means (treating each interview as an independent observation), followed by Tukey’s honestly significant difference (HSD) test for pairwise comparisons with family-wise error control.
2. *Linguistic features* for each linguistic feature, we conducted a one-way ANOVA with model as the factor, using per-question values as observations. Significant omnibus effects were followed by Tukey’s HSD for pairwise comparisons.
3. *Reliability* Cohen’s $\kappa$ was computed for each pair of annotators, and Fleiss’ $\kappa$ was computed across all three annotators, separately for each criterion.

### Alleviating possible concerns

#### Quality control and error handling

To ensure robustness and comparability across models, we implemented several safeguards.

1. *Standardized prompts* all interviewer models used identical persona descriptions, language settings, and prompt templates.
2. *Schema validation* the interviewer’s output was validated against a strict schema requiring specific fields (binary decision, justification, optional follow-up question). If validation failed, the orchestrator issued up to three constrained re-prompts requesting correction. After three consecutive failures on the same turn, the interview was aborted for that model to prevent accumulation of errors.
3. *Fixed interviewee* all follow-up questions were answered by the same GPT-4o-based interviewee agent to reduce response variability.
4. *Periodic summarization* the summarizer compressed reflection notes every 10 answers to stabilize context size and prevent memory overflow.
5. *Follow-up cap* a per-question limit (max_tailored_q = 5) prevented runaway loops where models might generate excessive follow-ups.

These controls ensured that observed differences reflected genuine model capabilities rather than artifacts of inconsistent orchestration.

#### Ethics, consent, and data handling

All baseline interviews were collected under informed consent for secondary computational analysis. Respondents were informed that their transcripts would be de-identified and used to evaluate automated interviewing systems. Transcripts were de-identified prior to processing, removing names, locations, and other identifying information while preserving linguistic and content features necessary for analysis.

This study analyzes LLM behavior in conducting interviews and does not provide clinical diagnoses or therapeutic interventions. All data were handled in accordance with institutional ethics guidelines and relevant data protection regulations. The study was conducted in accordance with the ethical standards of the Russian Psychological Society, as well as the Helsinki Declaration of 1975 and its later amendments. Informed consent was obtained from all individual participants to be included in the study. The study was approved by the Ethics Committee of the Institute of Psychology of Russian Academy of Science, approval number 6/12.

#### Reproducibility and code availability

To facilitate replication and extension of this work, we provide:

- complete prompts for all agents (generate_reasoning, reflect, generate_summary, interviewee_answer) in the “Appendix”.
- the orchestration graph implementation (LangGraph/Python) and evaluation code, to be released upon publication under an open-source license.
- an anonymized sample of interview turns sufficient to reproduce the statistical analyses.
- detailed annotation rubrics with examples (see “Appendix”).

The interview script is available upon request to researchers conducting similar studies. Complete baseline transcripts cannot be shared due to privacy considerations, but the provided sample demonstrates the data structure and analysis pipeline.

## Results

### Protocol compliance and model robustness

Of the six models evaluated, four—Claude Sonnet 4, Gemini 2.5 Pro, GPT-5 Chat, and Grok 4—successfully completed all ten standardized interviews without protocol violations. Qwen3-235B A22B occasionally returned malformed or incomplete responses but recovered through the retry mechanism and completed all ten interviews. DeepSeek Chat V3.1, however, exhibited persistent format compliance issues that prevented stable evaluation.

DeepSeek Chat V3.1 accumulated 45 schema validation errors across only three completed interviews, including ten instances where the model failed to produce the required decision marker or follow-up question format even after three retry attempts. The orchestrator halted evaluation after three interviews due to this instability. Because DeepSeek’s protocol violations resulted in systematically different execution conditions (e.g., aborted turns, missing follow-ups), its outcome statistics cannot be directly compared to the other five models. We report DeepSeek’s results for transparency but exclude it from comparative analyses and rankings.

### Throughput, latency, and questioning intensity

**Table 2:** Performance of LLM interviewers across ten standardized interviews. DeepSeek Chat V3.1 completed only three interviews; its statistics are reported for transparency but are not directly comparable.

Model	Time ([hh:]mm:ss[.t])			Average number of		
	Total	Averageper interview	Averageper follow-up	Follow-ups per interview	Follow-ups per main question	Characters per follow-up
Claude Sonnet 4	4:02:05	0:24:12	00:26.9	39.2	0.73	266.4
Gemini 2.5 Pro	9:40:03	0:58:00	01:04.4	31.8	0.59	247.5
GPT-5 Chat	1:28:22	0:08:50	00:09.8	19.4	0.36	184.8
Grok 4	15:51:54	1:35:11	01:45.8	45.7	0.85	292.5
Qwen3-235B A22B	24:07:20	2:24:44	02:40.8	26.8	0.50	240.2
DeepSeek Chat V3.1*	0:57:37	0:19:12	00:21.3	18.7	0.35	193.7

Table 2 presents performance metrics for all models across the ten standardized interviews. The models exhibited substantial variation in speed, efficiency, and questioning behavior.

#### Latency and throughput

GPT-5 Chat demonstrated the fastest performance, completing all ten interviews in 1:28:22 (mean 8 min 50 s per interview, 9.8 s per follow-up question). This represents near-real-time interactive capability suitable for live interviewing scenarios. In contrast, Qwen3-235B A22B required 24:07:20 total (mean 2 h 24 min per interview, 2 min 41 s per follow-up), making it impractical for synchronous interaction despite generating only moderate numbers of follow-ups. Grok 4 also showed substantial latency (15:51:54 total, 1 h 35 min per interview, 1 minute 46 s per follow-up), though this corresponded with the highest questioning intensity. Claude Sonnet 4 (4:02:05 total) and Gemini 2.5 Pro (9:40:03 total) occupied intermediate positions, offering practical throughput for research applications.

#### Questioning intensity

Grok 4 generated the most follow-up questions (mean 45.7 per interview, 0.85 per main question), reflecting a highly thorough but potentially exhausting interviewing style. Claude Sonnet 4 showed similarly high intensity (39.2 per interview, 0.73 per main question). GPT-5 Chat and DeepSeek Chat V3.1 adopted more selective approaches (19.4 and 18.7 per interview, respectively), asking fewer but potentially more necessary follow-ups. Gemini 2.5 Pro and Qwen3 fell in between (31.8 and 26.8 per interview).

#### Question length

Grok 4 also produced the longest follow-up questions (mean 292.5 characters), consistent with its syntactically complex style (see below). GPT-5 Chat and DeepSeek generated the most concise questions (184.8 and 193.7 characters), while other models ranged from 240–266 characters.

These patterns suggest a fundamental trade-off between speed and thoroughness: GPT-5 Chat optimizes for efficiency with brief, selective follow-ups, while Grok 4 prioritizes comprehensive coverage with lengthy, frequent questions at the cost of substantial latency.

**Table 3:** Operational token and cost profile of the evaluated models: token counts are mean prompt and completion tokens per API call, effective cost per follow-up is the total cost per interview divided by the average number of follow-ups per interview.

Model	Input tok./call	Output tok./call	USD/call	USD/interview	USD/follow-up
Claude Sonnet 4	3337.5	210.9	0.0132	2.95	0.075
Gemini 2.5 Pro	2156.6	1615.1	0.0188	3.88	0.122
GPT-5 Chat	2319.0	138.1	0.0043	0.75	0.039
Grok 4	2719.5	863.4	0.0210	5.03	0.110
Qwen3-235B A22B	2555.4	1778.7	0.0025	0.48	0.018
DeepSeek Chat V3.1${}^*$	2576.6	152.3	0.0012	0.21	0.011

${}^*$DeepSeek V3.1 completed only 3 interviews.

Table 3 shows the actual costs in terms of tokens and USD (at the time of access), making the cost-utility tradeoffs explicit. GPT-5 Chat combined the lowest latency among the fully comparable models with low end-to-end cost ($0.75 per interview, approx. $0.039 per realized follow-up), whereas Grok 4 and Gemini 2.5 Pro were the most expensive end-to-end ($5.03 and $3.88 per interview, respectively). Qwen3 was inexpensive per interview but limited by very high latency, so cost-efficiency and interactive usability do not always coincide.

### Expert evaluation of interviewer behavior

**Table 4:** Inter-rater reliability (Cohen’s $\kappa$ pairwise and Fleiss’ $\kappa$ across three raters).

Criterion	$\kappa _{12}$	$\kappa _{13}$	$\kappa _{23}$	Fleiss $\kappa$
Benevolence	0.92	0.95	0.92	0.93
Need for a follow-up	0.59	0.70	0.72	0.67
Context use	0.69	0.72	0.65	0.69
Openness of the question	0.86	0.88	0.81	0.85
Justified omission	0.87	0.94	0.86	0.89

Three expert psycholinguists annotated 1,658 follow-up questions on four quality criteria (benevolence, necessity, context-awareness, openness) and 1,275 instances of no follow-up on the “justified skip” criterion. Inter-rater reliability was high across all criteria (Table 4), with Fleiss’ $\kappa$ ranging from 0.67 (necessity) to 0.93 (benevolence), indicating substantial to almost perfect agreement.

**Fig. 5:**
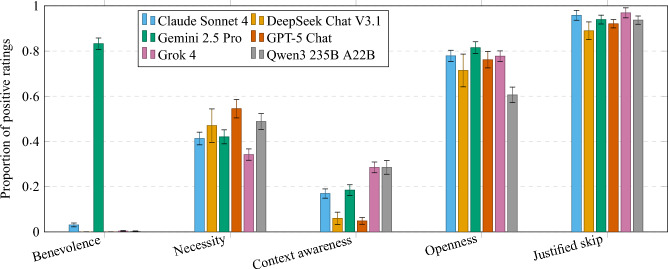
Expert judgments across models (mean $\pm 95\%$ CI; Wilson).

Figure 5 visualizes per-model mean scores; key findings are as follows.

1. *Benevolence (empathic tone).* Gemini 2.5 Pro substantially outperformed all other models, with expert annotators identifying empathic phrasing, such as “Thank you for sharing such a personal experience” or “I appreciate your openness in discussing this,” in a significantly higher proportion of its follow-ups. This advantage aligns with Gemini’s elevated use of intensifiers and second-person address. Other models showed moderate benevolence scores, with no significant differences among them.
2. *Necessity (warranted follow-ups).* GPT-5 Chat scored highest, reflecting its selective questioning strategy: by asking fewer follow-ups overall, it concentrated on cases where additional information was genuinely needed. Grok 4 scored lowest on this criterion, consistent with its very high questioning intensity (45.7 follow-ups per interview). Annotators noted that Grok frequently asked follow-ups even when prior answers were already detailed and complete. This suggests a quantity-quality trade-off: comprehensive coverage (Grok) versus selective precision (GPT-5).
3. *Context-awareness (integration of prior responses).* Grok 4 and Qwen3 demonstrated the strongest context use, frequently referencing information disclosed earlier in the interview. However, annotators noted that Grok sometimes over-relied on salient details in ways that felt repetitive or heavy-handed. In one extreme case, Grok invoked a respondent’s high neuroticism score 24 times within a single interview, often in contexts where annotators judged the reference awkward or unnecessary. Qwen3 showed similarly strong context integration without this issue, suggesting more balanced retrieval from long-term memory.
4. *Openness (non-leading questions).* All models performed well on this criterion, with mean scores above 0.80. Gemini 2.5 Pro achieved the highest score, while Qwen3 was slightly lower, though differences were modest. This suggests that contemporary LLMs generally avoid overtly leading phrasing when prompted appropriately, though subtle variations remain.
5. *Justified skip (appropriate omission of follow-ups).* When models chose not to ask a follow-up, this decision was almost always appropriate—mean scores ranged from 0.85 to 0.93 across models. Claude Sonnet 4 and Grok 4 showed slightly higher rates of justified skips, while DeepSeek was lower, but all models performed well. This indicates that the decision-making component of the interviewer agent functions reliably across architectures.

**Fig. 6:**
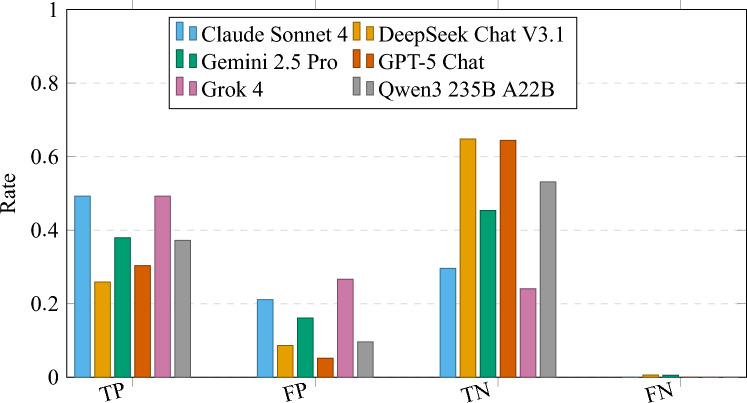
Decision quality for follow-up questions (TP/FP/TN/FN rates).

To assess how reliably each model decided whether to ask a follow-up, we compared its one-word decision (“ask” vs. “skip”) with expert judgments of Necessity and Justified skip. Figure 6 summarizes the resulting confusion matrix. Across all models, true negative rates dominate—most agents correctly recognized when no follow-up was needed—while false negative rates are minimal. Claude Sonnet 4 and Grok 4 achieved the highest true-positive ratios, Gemini 2.5 Pro and GPT-5 Chat are more conservative, preferring to skip marginal cases, which results in slightly lower recall but fewer unnecessary prompts. Overall, these results indicate that current LLM interviewers already make largely consistent “ask/skip” decisions, differing mainly in how aggressively they pursue additional questioning.

### Overall ranking across expert criteria

**Table 5:** Mean rank across expert criteria. Lower is better.

Model	Mean rank
Gemini 2.5 Pro	2.4
Grok 4	3.0
Claude Sonnet 4	3.2
GPT-5 Chat	3.6
Qwen3-235B A22B	3.6
DeepSeek Chat V3.1	4.2

To provide an aggregate assessment, we ranked models separately on each of the five expert criteria (lower rank = better performance), then computed the mean rank across criteria for each model (Table 5). Gemini 2.5 Pro achieved the best overall ranking (mean rank 2.4), driven primarily by its strength in Benevolence and solid performance across other dimensions. Grok 4 (3.0) and Claude Sonnet 4 (3.2) followed, balancing thoroughness and context-awareness against moderate necessity scores. GPT-5 Chat and Qwen3 tied (3.6 each), reflecting different strengths: GPT-5’s selective precision versus Qwen3’s structured context use. DeepSeek ranked last (4.2), though this reflects limited data from only three interviews.

### Linguistic style and syntactic complexity

**Table 6:** Distinctive linguistic markers of follow-up questions by model (selected features), showing the model mean and grand mean.

Claude Sonnet 4			Gemini 2.5 Pro			GPT-5 Chat		
Feature	Model	Grand	Feature	Model	Grand	Feature	Model	Grand
*Elevated features*								
N_pronn_pers_sec	2.673	2.176	N_pronn_pers_first	0.094	0.034	—		
N_repeated_words	10.388	8.105	N_pronn_pers_sec	2.601	2.176			
N_verb_aspect_imp	3.987	3.119	N_sconj	1.673	1.100			
N_verb_mood_ind	4.497	3.723	N_verb_mood_ind	4.223	3.723			
N_verb_root_tense_pres	0.967	0.568	N_verb_root_tense_past	1.403	0.666			
N_verb_tense_pres	2.372	1.788	N_verb_tense_past	2.903	2.125			
N_verb_voice_act	5.194	4.473	Intensifiers	3.890	2.701			
*Suppressed features*								
—			N_conj	2.041	2.886	N_adj	1.897	2.983
			Mean words/sent	13.326	25.983	N_sconj	0.531	1.100
			Mean word length	5.305	5.526	N_verb_aspect_perf	1.624	2.456
			Mean syntactic depth	4.043	5.892	N_verb_root_aspect_perf	0.149	0.667
			Min syntactic depth	2.638	5.243	N_verb_root_voice_act	0.974	1.415
						N_verb_tense_past	1.222	2.125
						Total words	28.134	37.958
Grok 4			Qwen3-235B A22B			DeepSeek Chat V3.1*		
Feature	Model	Grand	Feature	Model	Grand	Feature	Model	Grand
*Elevated features*								
N_adv	4.009	3.040	Mean word length	5.815	5.526	—		
N_advcl	0.567	0.389						
N_cconj	3.059	2.382						
N_conj	3.786	2.886						
N_pronn_pers_third	0.558	0.342						
N_relative	1.245	0.980						
N_verb_mood_imp	0.761	0.334						
Total words	43.770	37.958						
Max words/sent	43.155	29.128						
Max syntactic depth	8.175	6.582						
Mean words/sent	43.001	25.983						
Mean syntactic depth	8.142	5.892						
Min syntactic depth	8.109	5.243						
*Suppressed features*								
N_verb_root_aspect_imp	0.284	0.882	Intensifiers	1.279	2.701	N_adj	1.964	2.983
N_verb_root_mood_ind	0.315	1.227				N_cconj	1.554	2.382
N_verb_root_tense_past	0.125	0.666				Total words	28.161	37.958
N_verb_root_tense_pres	0.195	0.568				Max words/sent	17.714	29.128
N_verb_root_voice_act	0.982	1.415				Mean words/sent	13.824	25.983
						Min syntactic depth	2.964	5.243

*DeepSeek statistics based on only three completed interviews; not directly comparable

We analyzed 1658 follow-up questions using morpho-syntactic features and psycholinguistic categories from a Russian LIWC-like dictionary. One-way ANOVAs identified significant between-model effects for 63 of 95 features tested ($p < 0.05$); 32 features showed no significant differences (e.g., superlative adjectives, discourse markers, future tense, many affect categories). Post-hoc Tukey HSD tests revealed at least one significant pairwise difference for 52 features, while 43 features showed significant omnibus effects but no significant pairwise contrasts surviving family-wise error correction.

Table 6 summarizes the most distinctive linguistic markers for each model—features that showed at least three significant pairwise contrasts in Tukey tests and exhibited model means within 0.2 standard deviations of the global maximum or minimum across models. These linguistic features reveal some interpretable stylistic profiles.

1. *GPT-5 Chat* (and DeepSeek when functional) produced minimalistic questions: below-average counts for adjectives, subordinating conjunctions, perfective verbs, past tense, and total words. This concise style aligns with GPT-5’s efficiency-first approach.
2. *Grok 4* occupied the opposite extreme, generating syntactically elaborate follow-ups with elevated coordinating conjunctions, adverbs, adverbial clauses, third-person pronouns, imperative mood, and maximum syntactic depth—but suppressed verb roots in several morphological categories. Questions averaged 43 words with mean syntactic depth exceeding eight levels, compared to a grand mean of 26 words and depth 5.9. This complexity may enhance thoroughness but risks overwhelming respondents.
3. *Claude Sonnet 4* emphasized present-tense, imperfective, and active-voice constructions with frequent second-person address, creating a dialogic, immediate tone focused on the respondent’s current experience. The model also showed elevated word repetition within questions, potentially reinforcing key concepts.
4. *Gemini 2.5 Pro* used more first- and second-person pronouns, past tense, subordinating conjunctions, and intensifiers, while producing shorter sentences and shallower syntax than Grok or Claude. This combination suggests a more conversational, reflective style that acknowledges shared understanding (first person) while focusing attention on the respondent (second person) and their past experiences.
5. *Qwen3-235B A22B* favored longer words (mean 5.8 vs. 5.5 characters overall) while avoiding intensifiers, producing a more formal, structured register.

These stylistic differences are not merely cosmetic; they correlate meaningfully with human judgments of interviewer quality (see below), suggesting that linguistic choices directly impact perceived empathy, appropriateness, and effectiveness.

### Summary of trade-offs and model profiles

The evaluation reveals distinct profiles suited to different research priorities:

1. *Speed vs. thoroughness.* GPT-5 Chat delivers near-real-time performance (9.8 sec/follow-up) with concise, mostly necessary questions, making it well-suited for cost- and time-constrained deployments or screening scenarios. Grok 4’s exhaustive questioning (45.7 follow-ups/interview) extracts maximum detail but requires substantial time (1:46 min/follow-up) and risks respondent fatigue. Occasional over-contextualization (e.g., excessive references to personality traits) further limits suitability for sensitive domains.
2. *Interpersonal tone.* Gemini 2.5 Pro’s empathic, benevolent style—evidenced by highest Benevolence scores and elevated intensifiers in linguistic analysis—is particularly appropriate for interviews on sensitive autobiographical topics (trauma, illness, identity) where rapport matters. Its conversational use of first-person pronouns (acknowledging shared understanding) and past-tense focus (reflecting on experiences) creates a warm, supportive interviewing presence.
3. *Context integration and structure.* Qwen3 demonstrated strong context-awareness and a more formal register (longer words, fewer intensifiers, lower openness), suitable for structured protocols requiring systematic coverage. However, its latency (2:41 min/follow-up) limits interactive use. Claude Sonnet 4 offered balanced performance with a dialogic, present-focused style and practical throughput (27 sec/follow-up), making it a versatile choice across interview types.
4. *Reliability.* DeepSeek’s format instability disqualifies it under the current protocol, highlighting the importance of schema compliance for production systems.

In total, model selection should align with study goals: prioritize GPT-5 for efficiency, Gemini for empathy-sensitive contexts, Grok for maximum thoroughness (with caution), Claude for balanced versatility, and Qwen3 for structured protocols where latency is acceptable.

## Discussion

In this study, we have introduced a modular LLM agent for conducting adaptive psychological interviews and established a principled framework for evaluating interviewer behavior. By standardizing interview context, orchestration, and follow-up responses across six state-of-the-art models, we isolated genuine differences in interviewing capability and revealed systematic trade-offs across speed, thoroughness, empathy, and context understanding.

We have arrived at three main findings. First, current LLMs differ substantially not only in latency but also in the quality dimensions that matter for qualitative research: benevolence, necessity, context-awareness, and openness. Gemini 2.5 Pro has an empathic tone that would work well for sensitive autobiographical interviews, while GPT-5 Chat’s selective, efficient approach suits high-throughput screening. Grok 4’s exhaustive questioning extracts maximum detail but risks overwhelming respondents and occasionally overdoes the context. Second, linguistic markers such as person pronouns, tense and aspect, intensifiers, and syntactic complexity align meaningfully with human judgments, suggesting that stylistic choices directly shape perceived interview quality. Third, schema compliance remains critical: DeepSeek’s format instability rendered it basically unusable under the current protocol, and reliable structured output remains an important criterion for production systems in many domains, including psychological interviewing.

These results lead to practical implications for deploying LLM interviewers in psychological experiments. We advise researchers to match model capabilities with study goals: efficiency-critical applications (large-scale screening, preliminary assessments) favor GPT-5; empathy-sensitive contexts (trauma, identity, illness narratives) favor Gemini; comprehensive interviewing with a tolerance for latency suits Grok or Claude. Our five-criterion rubric and linguistic profiling pipeline offer a reusable audit tool, enabling practitioners to benchmark new models or fine-tuned variants before deployment and to detect behavioral drift over time. Since frontier LLMs evolve rapidly, model-specific rankings reported here should be interpreted as a time-stamped benchmark rather than a permanent leaderboard, and the exact ordering we have observed is unlikely to remain stable across subsequent model revisions. The more durable contribution is the evaluation protocol itself: a controlled way to compare models on benevolence, necessity, context-awareness, openness, schema compliance, latency, and cost under matched interview conditions. In that sense, the present study provides both a snapshot of the models available during the study window and a template for repeated re-benchmarking as new versions appear.

Several limitations qualify these findings. First, our evaluation used Russian-language interviews with a specific cultural and demographic context; generalization to other languages, interview structures, or respondent populations requires empirical validation. We expect the expert annotation criteria themselves (benevolence, necessity, context-awareness, openness, and justified skip) to transfer readily across languages because they target pragmatic properties of interviewer behavior rather than Russian-specific morphology. In contrast, linguistic-feature analysis is partly language-bound: Russian aspectual and inflectional cues, as well as dictionary-based intensifier counts, will not map one-to-one to English or other languages. Therefore, a multilingual validation would rerun the same protocol on matched baseline interviews in at least one additional language (e.g., English), apply the same expert rubric, and replace the linguistic-feature set with language-appropriate parsers and lexicons; this kind of cross-lingual generalization requires further large-scale experiments and are left as future work. Second, the fixed LLM interviewee (GPT-4o) standardizes follow-up responses but introduces a dependency: model rankings might shift if interviewee responses were different in style or quality. Third, our annotation focuses on interviewer behavior rather than downstream outcomes (e.g., diagnostic accuracy, therapeutic alliance, respondent satisfaction). Fourth, we evaluated text-only interactions; voice-based or multimodal interviews (incorporating prosody, facial expressions, pauses) may introduce additional important dimensions for analysis, but are also left for future work.

Beyond these methodological extensions, we propose several novel substantive questions for future work. How do model-specific interviewing styles interact with respondent characteristics (age, education, cultural background, personality traits)? Are there systematic biases in which types of disclosures different models elicit or suppress? Can interviewer agents be fine-tuned or steered via prompting to adopt specific therapeutic modalities (e.g., motivational interviewing, cognitive-behavioral techniques) while maintaining the quality criteria we measured? How do repeated interactions over multiple sessions affect coherence, memory, and rapport? Addressing these questions may require longitudinal studies, diverse respondent samples, and integration with domain-specific outcome measures.

Our findings also intersect with ongoing debates about LLMs in psychological research. We agree with concerns that LLMs should not be treated as faithful simulators of human psychology (they are very different intelligences than humans) but rather as linguistic tools with specific affordances and validity conditions. The interviewer agent we developed is not a replacement for human interviewers in contexts requiring nuanced judgment, ethical oversight, or therapeutic presence. Instead, it is a scalable complementary tool useful for preliminary screening, large-scale data collection, standardizing interview protocols, or helping human researchers by handling routine questioning while flagging cases for expert follow-up. Responsible deployment requires transparency (disclosing LLM use to participants), ongoing monitoring (detecting biases or harmful outputs), and human oversight (especially in clinical settings).

The interviewer agent architecture we introduced—fusing reasoning and question generation, maintaining dual memory, and enforcing structured output—proved stable across most models and offers a foundation for further development. Extensions might include active learning (adapting question strategy based on accumulating responses), multi-turn negotiation (clarifying ambiguous answers before moving on), or integration with external knowledge bases (e.g., referencing prior session notes, medical records, or validated assessment instruments). The modular design facilitates such extensions while preserving the controlled evaluation framework.

In conclusion, this work demonstrates that contemporary LLMs can already approximate several core functions of adaptive interviewing under controlled, text-only conditions when properly orchestrated and evaluated, but that meaningful differences in empathy, thoroughness, efficiency, and stylistic sophistication exist among models. Our controlled evaluation protocol and principled rubric provide a replicable methodology for auditing interviewer agents, guiding model selection, and advancing the responsible integration of LLMs into psychological research. As these technologies improve further, rigorous domain-informed evaluation, focused not just on task completion but on the quality of human interaction, will be essential to realizing their potential.

## Data Availability

All data used in this study has been made available at the IEEE Dataport^48^: https://dx.doi.org/10.21227/kbnf-gs17.

## A Prompts for large language model (LLM) agents

### A.1 Prompt for evaluating response completeness and generating clarifying questions

This prompt enables the interviewer LLM agent to assess the completeness of a respondent’s answer to the main question. If the answer is deemed incomplete, the prompt initiates generation of a targeted clarifying question to collect missing information.

Role: Interviewer.

{Meta-information:

Language: Russian

Interviewer description (Isabella): friendly and curious}

* Goal: Determine whether the respondent’s answers CONTAIN KEY INFORMATION

necessary for a COMPLETE AND DETAILED answer to the MAIN QUESTION, even if

the answer contains ADDITIONAL and REDUNDANT information.

Main question: [target_question]

Respondent’s last answer: [user_answer]

Current conversation transcript: [short_term_memory]

Previous self-reflection notes: [reflection_notes]

—

INSTRUCTIONS FOR LLM-AGENT:

Part 1: Assessment and Reasoning

1. Evaluate the interview progress: Think step by step:

                * What information has been obtained from the respondent so far

                   (from “Last Answer”, “Conversation Transcript” and

                   “Self-Reflection Notes”)?

                * What exactly is the “main information” necessary for a complete

                    and detailed answer to “[target_question]”?

                * Was this “main information” provided in the last answer and

                   current conversation transcript? Consider that the presence of

                   additional or redundant information does not hinder achieving

                   the goal if the main information is present.

                * Is the information provided by the respondent sufficient to be

                    considered a complete and detailed answer to the main question?

2.         Write a brief assessment (3-4 sentences): Summarize whether the

            interview goal is achieved, based on your reasoning. Explain why

            you reached this conclusion.

Part 2: Determination of Goal Achievement (ONE WORD)

Answer whether the interview goal has been achieved using ONLY ONE WORD:

*       ’yes’: If the last answer and answers from the conversation transcript

         contain the necessary key information for the main question, or if

         the answer explicitly or implicitly demonstrates refusal to answer

         the question.

*        ’no’: If the last answer and answers from the conversation transcript

         do not contain the necessary key information for the main question,

         are insufficiently detailed, and are not an explicit or implicit

         refusal to answer. Do not consider redundancy or irrelevance of

         additional information.

Part 3: Formulation of Clarifying Question (If Goal Not Achieved)

If the goal is not achieved (answer ’no’), formulate a CLARIFYING QUESTION.

* The question should directly rely on missing information or unclear

      points identified in your reasoning.

* The question should be maximally specific and aimed at obtaining

      concrete information not indicated in the “Conversation Transcript”

      and “Self-Reflection Notes”.

* The question should differ from those already asked in the “Current

      Dialogue Transcript”.

* Avoid questions that can be answered ’yes/no’, unless this leads to

      obtaining necessary detailed information. The goal is to obtain new,

      missing details.

—

FORMAT OF YOUR FINAL ANSWER:

Please strictly adhere to one of the following two formats:

Case 1: Interview goal achieved

Interview progress assessment: [Your 3-4 sentence assessment]

Interview goal achieved:

yes

Case 2: Interview goal NOT achieved

Interview progress assessment: [Your 3-4 sentence assessment]

Interview goal achieved:

no, additional question: [Your clarifying question]

### A.2 Prompt for generating reflective notes based on respondent answers

This prompt is used to synthesize brief, objective facts and observations about the respondent based on their answers. This helps the agent form a knowledge model about the respondent and adapt further course of dialogue.

ROLE: You are an analyst and interviewer, your task is to create brief

and clear reflection points based on the current dialogue. These points

should help in further assessment of the respondent and in formulating

future questions.

DIALOGUE CONTEXT:

- Main question: [target_question]
- Respondent’s last answer: [user_answer]
- Previous reflection notes (to avoid duplication): [reflection_notes]

INSTRUCTIONS:

1. Carefully analyze the respondent’s last answer in the context of

      the main question.

2. Formulate one to two NEW observations or facts about the respondent.

      If the answer is short, limit yourself to one observation.

3. New facts and observations should be evident EXCLUSIVELY FROM THE

      DIRECT TEXT OF THE RESPONDENT’S ANSWER.

4. Generate only dry facts, observations. Evaluative judgments,

      assumptions and hypotheses based on the answer are prohibited.

5. Carefully analyze previous reflection notes to avoid duplication

      of information. Make special focus on the last 10 points, as they

      may contain information you are about to add.

6. Present your conclusions as a bulleted list.

7. Each individual observation should be objective and informative,

      length should not exceed 15 words.

### A.3 Prompt for summarization and compression of reflective notes

This prompt is designed for summarization and compression of accumulated reflective memory. It removes duplications and combines semantically similar points, which increases the efficiency of the agent’s long-term memory.

Prompt of the model that generated the notes:

INSTRUCTIONS:

1. Carefully analyze the respondent’s last answer in the context of

      the main question.

2. Formulate one to two NEW observations or facts about the respondent.

      If the answer is short, limit yourself to one observation.

3. New facts and observations should be evident EXCLUSIVELY FROM THE

      DIRECT TEXT OF THE RESPONDENT’S ANSWER.

4. Generate only dry facts, observations. Evaluative judgments,

      assumptions and hypotheses based on the answer are prohibited.

5. Carefully analyze previous reflection notes to avoid duplication

      of information. Make special focus on the last 10 points, as they

      may contain information you are about to add.

6. Present your conclusions as a bulleted list.

7. Each individual observation should be objective and informative,

      length should not exceed 15 words.

Current version of notes:

reflection_notes

Analyze the current version of notes for duplication.

1. Remove duplicate points if they exist.

2. Combine semantically similar points into one, if possible without

      loss of information.

3. The final answer should be at least twenty percent shorter than

      the initial version.

4. Do not exclude clear facts concerning profession, family composition,

      gender, age, education, etc.

Output the final version of notes as a bulleted list, without any

additional comments or explanations.

### A.4 Prompt for personalized generation of answers to follow-up questions

This prompt serves to create respondent answers that are not only logically correct but also correspond to a given psychological profile. This allows simulation of realistic and consistent human behavior in dialogue.

Role: Interviewee.

Meta-information:

Language: Russian

Personality traits of the interviewee according to the Big Five model:

extraversion: level;

agreeableness: level;

neuroticism: level;

conscientiousness: level;

openness to experience: level.

—

Goal: Formulate a COMPLETE, LOGICAL AND PERSONALITY-APPROPRIATE ANSWER

TO THE QUESTION.

—

Given question: [question]

Current conversation transcript: [short_term_memory]

—

INSTRUCTIONS:

1. Analyze the “Given question” and study the “Current Conversation

      Transcript” to understand the context and gather relevant information.

2. Formulate an answer that:

* Directly answers the question and corresponds to the psychological

      traits of the interviewee (for example, high level of conscientiousness

      = structured answer; low neuroticism = calmness).

* Relies on information from the transcript, avoiding repetitions

      or contradictions.

* Is logical, coherent, complete and avoids excessive verbosity,

      unless justified by personality.

* Adheres to the style and tone characteristic of the transcript.

IMPORTANT: Focus on providing relevant and accurate information.

## B Interview questions

1. To start, tell me where you’re from? Where did you grow up and what was that place like?
2. Remembering your school years, what kind of student were you?
3. Were there teachers who influenced you? If yes, in what way? What were they like?
4. What subject in school was your favorite and why?
5. What subject did you dislike the most and why?
6. Remembering childhood, who were your heroes (role models) and why?
7. What did you dream of becoming when you grew up and why?
8. What did you dream about and what plans did you have after finishing school? What influenced their formation?
9. If you had complete freedom of choice, what would be your dream job and why?
10. Is there something you’ve dreamed about throughout your life and continue to strive for?
11. Let’s move to your childhood—how would you describe the personalities of your family members you grew up with? For example, what were your parents and/or sisters and brothers like?
12. How are you similar to or different from your parents and/or siblings?
13. What was the best thing about your childhood?
14. And what do you consider the worst moments of childhood?
15. Moving to another topic—what was your first paid job? How old were you then? (If not applicable, simply answer that you didn’t work)
16. What do you do now professionally? Why did you choose this profession?
17. Please describe your typical work day.
18. What do you like and dislike about your current job?
19. What human qualities in you do your friends especially value?
20. Friendship sometimes requires sacrifices and efforts. Are there cases when you have to overcome yourself to be a good friend? What exactly do you struggle with in such situations?
21. Now more general questions: what are you most proud of in life?
22. What hobbies (interests) or other interests do you have?
23. What scares you now?
24. And what scared you most in childhood?
25. Tell about a life situation when you didn’t know if you would cope. How did you overcome this challenge (trial)?
26. Some say that in such moments they made a conscious decision, and others—that “everything worked out by itself”. How was it for you?
27. In difficult times some say they cope with the help of smoking or alcohol. How about you?
28. Tell about a case in the last year when it was hard for you or you experienced emotional difficulties.
29. Some say that religion or spirituality are important to them, others—that they are not. What place does religion occupy in your life?
30. Now let’s move to the present. Tell about your family, about people who are important to you. Do you have a partner (life companion), children?
31. Now we would like to know more about your health. How do you assess your health?
32. How important is it for you to take care of your health? What do you try to do to maintain your health? What do you try to avoid?
33. There are different attitudes toward vaccination in society. What do you think about this? Do you get vaccinated? How safe do you consider it?
34. Have you or your family used alternative treatment methods?
35. How would you describe your relationships at work? (With management, with colleagues?)
36. How predictable is your work schedule? How flexible is it?
37. Some say that because of children it’s difficult to work. How is it for you?
38. How would you describe the attitude toward money that was characteristic of your parents? And what do money mean to you personally?
39. How do you generally assess your financial situation?
40. Were there cases when you experienced financial difficulties. What did you do to cope with such situations?
41. We all have hopes for the future. Imagine your life in a few years. What would you like to remain the same? What would you like to change?
42. What is most valuable to you in life?
43. Name three major news events that happened during your lifetime?
44. Name 3-4 important personality traits of a candidate you would vote for in elections?
45. If you could solve one—and only one—world problem, what would it be and why?
46. Tell the story of your life. Start from the very beginning—from childhood, then education, family and relationships, and any important events in your life.
47. Please recall and describe an episode from your childhood that could explain what kind of person you are, reveal something important in you. Specify why you remembered this particular episode? What qualities of yours, in your opinion, are connected with this event?
48. Please recall and describe an episode from your youth that shows your personality, in which its central properties manifested. Tell what aspects of your personality are captured in this episode.
49. Please recall and describe an event from the recent past where you were maximally “yourself”. Explain how your self is revealed in this memory.
50. Imagine and describe in maximum detail an event from your future that, in your opinion, will best reflect your essence and your values. How will the most important thing in you be reflected in this event?
51. In your opinion, which traits of your character and your values will change in 10 years? And which will remain the same?
52. What changes in people throughout life, and what remains unchanged?
53. Everyone has victories and defeats in life. Please describe a situation in which you experienced failure and strong disappointment. What goals did you set for yourself in this situation, what seemed especially important to you?
54. What did you do to achieve these goals? What lessons did you learn from this experience? Were there later situations in life in which this experience was useful?

## C Criteria for evaluating follow-up questions (with examples)

### C.1 Friendliness of the question

This criterion concerns the general tone of follow-up questions asked. The friendliness of a question is evidenced by the following signs: presence in the follow-up question of positive evaluations of the respondent’s judgments, actions, goals, wishes, etc. (“Thank you for the answer!”, “Very interesting!” etc.); expression of empathy by the interviewer toward the respondent.

**Examples of “Yes” (presence of friendliness)** (here and below, parts of the interviewer’s remark relevant to the criterion are shown in italics):

- Example 1. Follow-up question (FQ): *Interesting, thank you!* Could you describe this neighborhood in a bit more detail? What exactly made it “ordinary” in your view and what do you remember most from childhood?
- Example 2. FQ: You mentioned that you wanted to become a psychiatrist to help people. *This is a very noble goal.* And if we look at this through the lens of what you said earlier—about your interest in complex systems and admiration for how researchers brought structure to chaos—how did this side of you manifest in your desire to become a psychiatrist?
- Example 3. FQ: *Thank you for sharing such a personal and deep memory.* You mentioned that this experience taught you care, but was also traumatic. In your opinion, how did this necessity at age 12 to take on such enormous responsibility and face the chaos of illness influence your adult striving to systematize, analyze and control everything?

**Examples of “No” (absence of friendliness)**:

- Example 1. FQ: Can you tell more about what your neighborhood was like? What memories do you have associated with this place?
- Example 2. FQ: Why specifically is work as an art authentication expert your dream job, and what specific aspects of this profession inspire you?
- Example 3. FQ: How old were you when you started helping your sister with blog design?

### C.2 Necessity of asking the question

This evaluates how necessary the follow-up question is, whether it is excessive given that the answer to the main question was sufficiently complete. The content of the follow-up question is correlated with the completeness of the answer to the main (and to the follow-up question—in the case when the second follow-up question is being evaluated).

**Variants of incomplete answer requiring a follow-up question**: in the question the respondent is asked to answer about *a* and *b*, but they answer only about *a*, thus the interviewer should ask again about *b*; the answer is too brief and uninformative. The interviewer should clarify the meaning of what was said, obtain more information on the topic of the question.

**Examples of “Yes” (the main question received a brief, general or incomplete answer, making a follow-up question necessary)**:

- Example 1. Main Question (MQ): To start, tell me where you’re from? Where did you grow up and what was that place like? Main Answer (MA): I grew up in an ordinary residential neighborhood in northern Moscow. FQ: Can you tell more about what your neighborhood was like? What memories do you have associated with this place?
- Example 2. MQ: Moving to another topic—what was your first paid job? How old were you then? (If not applicable, simply answer that you didn’t work) MA: The first paid job was unofficial, I helped my sister with her work, and she transferred me some money... Small earnings, but pleasant, because it was related to blog design. FQ: How old were you when you started helping your sister with blog design?
- Example 3. MQ: If you had complete freedom of choice, what would be your dream job and why? MA: now I want to work in school as a math teacher or tutor. but more in school, with small classes FQ: Considering your striving to support others (like your psychologist mother and your math teacher) and spiritual values (through the musician’s example), what specific aspects of working as a math teacher in a small class make it your dream job, realizing these aspirations more fully than the profession of psychologist?

**Examples of “No” (a sufficiently complete answer was received to the main question and there is no need for a follow-up question, but it was still asked)**:

- Example 1. MQ: Were there teachers who influenced you? If yes, in what way? What were they like? MA: The math teacher was completely unemotional, but nevertheless she showed that if you answer clearly and quickly to the question posed, everything will be fine. It was impossible to establish contact with her, but it was necessary to answer as in the army. This taught me to formulate my thoughts clearly and quickly. FQ: Can you tell about other teachers who influenced you, and how exactly this influence manifested?/FQ: Could you describe more specifically how the experience of interacting with this teacher or other educators influenced your life principles, choice of profession or attitude toward learning in the future?
- Example 2. MQ: What subject in school was your favorite and why? MA: I loved algebra and geometry. Because in these subjects there is logic, you need to know theorems, rules and then everything is solved quite easily. FQ: Can you tell in more detail what exactly attracted you in algebra and geometry, how you felt when studying these subjects?

### C.3 Contextuality of the question

This criterion concerns the extent to which the interviewer takes into account information obtained when answering previous questions (main and follow-up) and uses it when formulating the follow-up question. In the text of the follow-up question there may be both a clear indication by the interviewer that they are using information from earlier answers (You mentioned that...; ), and more veiled forms of accounting for context (This is very consonant with your childhood interest in exploring unstructured spaces; ...your adult striving to systematize, analyze and control everything...).

*Examples of “Yes” (in the text of the follow-up question, information obtained from answers to previous questions is taken into account)*:

- Example 1. You mentioned that you liked subjects with clear logic, like algebra, and subjects where you can find non-obvious connections, like literature. What exactly in school history, in your opinion, prevented you from finding the same structure or interesting interrelations that would engage you?
- Example 2. FQ: You named travelers and discoverers, such as Nansen and Amundsen. This is very consonant with your childhood interest in exploring unstructured spaces. Tell me, what specific qualities or actions of theirs evoked your admiration? What exactly made them heroes in your eyes?
- Example 3. MQ: What subject did you dislike the most and why? MA: History, because I was never particularly interested in it. FQ: You mentioned that you liked subjects with clear logic, like algebra, and subjects where you can find non-obvious connections, like literature. What exactly in school history, in your opinion, prevented you from finding the same structure or interesting interrelations that would engage you?

*Examples of “No” (information obtained from answers to previous questions is not used in the follow-up)*:

- Example 1. MQ: What human qualities in you do your friends especially value? MA: Kindness, intelligence, sensitivity, subtle perception FQ: Can you tell about specific situations in which your friends notice and value these qualities in you?
- Example 2. MQ: Let’s move to your childhood—how would you describe the personalities of your family members you grew up with? For example, what were your parents and/or sisters and brothers like? MA: My mother is somewhat infantile. Father is dependent on alcohol. Grandmothers have quite rigid characters. FQ: Can you tell how the characters of your parents and grandmothers influenced your childhood and the formation of your personal qualities?

### C.4 Openness of the question

The interviewer in the follow-up question offers their own answer variants, thereby to a certain extent influencing the respondent’s expression of their own opinion. If such “hints” are absent in the follow-up question, then by this criterion it receives a “Yes” evaluation, if “hints” are present, then—“No”.

*Examples of “Yes”*:

- Example 1. MQ: What human qualities in you do your friends especially value? MA: Kindness, intelligence, sensitivity, subtle perception FQ: Can you tell about specific situations in which your friends notice and value these qualities in you?
- Example 2. MQ: If you could solve one—and only one—world problem, what would it be and why? MA: If I really could solve absolutely any problem, I would choose something global, so that changes in this would positively influence the solution of other problems. And something from which people really suffer greatly. Something like problems of wars, hunger, social inequality. FQ: You named several very serious problems—wars, hunger, social inequality—and explained that you would like to choose something global. If you had to stop at only one of them, which would you choose and why exactly does it seem to you primary compared to the others?

**Examples of “No”**:

- Example 1. MQ: Friendship sometimes requires sacrifices and efforts. Are there cases when you have to overcome yourself to be a good friend? What exactly do you struggle with in such situations? MA: I sometimes fear I’ll snap if I internally disagree with a friend. FQ: This is a very understandable internal struggle. You previously told how you help friends, applying your analytical approach. Tell me, does this fear of “snapping” usually arise when you see that a friend is making an illogical or chaotic, from your point of view, decision? And what exactly do you restrain in yourself at this moment—the desire to give a direct, possibly harsh assessment of the situation?
- Example 2. MQ: What scares you now? MA: Loss of motivation for movement and development FQ: Fear of not realizing your ambitious goals, or is this a deeper fear of stopping and succumbing to that very entropy which you’ve resisted all your life?

### C.5 Justification for skipping the follow-up question

This criterion concerns how justified the absence of a follow-up question is. Skipping a follow-up question is considered justified when the respondent gave complete answers to all questions asked within the framework of the Main question.

*Examples of “Yes” (absence of follow-up question is justified)*:

- Example 1. MQ: To start, tell me where you’re from? Where did you grow up and what was that place like? MA: I’m from the city of Snezhinsk, which is located in the Southern Urals. I spent all my childhood in this city before entering MSU. A closed city, a safe place to freely walk around the city as a child, not fearing the dangers of a big city. In high school my teacher called this city a “greenhouse”, and us “plants in it”, who grew up in favorable conditions, not knowing other life. In part I now agree with this.
- Example 2. MQ: What subject in school was your favorite and why? MA: I loved algebra and geometry. Because in these subjects there is logic, you need to know theorems, rules and then everything is solved quite easily.
- Example 3. MQ: Were there teachers who influenced you? If yes, in what way? What were they like? MA: The math teacher was completely unemotional, but nevertheless she showed that if you answer clearly and quickly to the question posed, everything will be fine. It was impossible to establish contact with her, but it was necessary to answer as in the army. This taught me to formulate my thoughts clearly and quickly.

*Examples of “No” (follow-up question is required)*:

- Example 1. MQ: How are you similar to or different from your parents and/or siblings? MA: I am completely different from them. I always strive for development, I never rest on my laurels.
- Example 2. MQ: What subject did you dislike the most and why? MA: History, because I was never particularly interested in it.
- Example 3. MQ: Remembering childhood, who were your heroes (role models) and why? MA: My heroes were travelers and discoverers: Fridtjof Nansen, Amundsen. People who went on expeditions to the Arctic and Antarctic.
